# Influence of combined functional resistance and endurance exercise over 12 weeks on matrix metalloproteinase-2 serum concentration in persons with relapsing-remitting multiple sclerosis – a community-based randomized controlled trial

**DOI:** 10.1186/s12883-019-1544-7

**Published:** 2019-12-06

**Authors:** Sebastian Proschinger, Niklas Joisten, Annette Rademacher, Marit L. Schlagheck, David Walzik, Alan J. Metcalfe, Max Oberste, Clemens Warnke, Wilhelm Bloch, Alexander Schenk, Jens Bansi, Philipp Zimmer

**Affiliations:** 1 Department for Molecular and Cellular Sports Medicine, Institute for Cardiovascular Research and Sports Medicine, German Sport University Cologne, Cologne, Germany; 2 Department of Neurology, University of Cologne, Faculty of Medicine and University Hospital Cologne, Cologne, Germany; 3 Deparment of Neurology, Kliniken-Valens, Rehabilitationsklinik-Valens, Taminaplatz 1, 7317 Valens, Switzerland; 4 Department of Exercise and Health, Institute of Sports Science, Leibniz University Hannover, Hannover, Germany

**Keywords:** Relapsing-remitting multiple sclerosis, Matrix metalloproteinase-2, Functional exercise, Immune homeostasis, Kynurenine pathway, Inflammation

## Abstract

**Background:**

The relevance of regular moderate to intense exercise for ameliorating psychomotor symptoms in persons with multiple sclerosis (pwMS) is becoming increasingly evident. Over the last two decades, emerging evidence from clinical studies and animal models indicate immune regulatory mechanisms in both periphery and the central nervous system that may underlie these beneficial effects. The integrity of the blood-brain barrier as the main structural interface between periphery and brain seems to play an important role in MS. Reducing the secretion of proteolytic matrix metalloproteinases (MMP), i.e. MMP-2, as disruptors of blood-brain barrier integrity could have profound implications for MS.

**Methods:**

In this two-armed randomized controlled trial 64 participants with relapsing-remitting MS (RRMS) (EDSS 0–4.0) will be allocated to either an intervention group or a passive wait list control group. The intervention group will perform 60 min of combined functional resistance and endurance exercises 3x per week over a period of 12 weeks in a community-based and publicly available setting. Changes in serum concentration of MMP-2 will be the primary outcome. Secondary outcomes are numbers of immune cell subsets, soluble (anti-) inflammatory factors, physical capacity, cognitive performance, physical activity behavior, gait performance, and patient-reported outcomes. All outcome measures will be assessed at baseline and after week 12 with an additional blood sampling before, during and immediately after a single training session in week 6.

**Discussion:**

To our knowledge, this will be the first RCT to investigate both the acute and chronic effects of a community-based intense functional resistance and endurance exercise regimen in persons with RRMS. Combining analysis of biological and cognitive or psychological outcomes may provide a better understanding of the MS-specific symptomology.

**Trial registration:**

DRKS00017091; 05th of April, 2019; International Clinical Trials Registry Platform.

## Background

Accumulating evidence over the past two decades underlines the role of a dysregulated immune system in the pathophysiology and progression of multiple sclerosis (MS) [1]. The broad immunoregulatory effects of regular exercise is becoming more evident and is now recommended as an adjunct therapy for people suffering from chronic (neuro-)inflammatory diseases, such as MS [2–4]. Evidence-informed physical activity guidelines for persons with MS (pwMS) have already been developed [5], but less than 30% of pwMS meet the recommended guidelines for moderate to vigorous exercise [6]. A perturbed peripheral immune homeostasis indicated by the imbalance of T cell subsets seems to be a major disease-specific characteristic [7, 8]. Interestingly, there might be a proposed intensity-dependent effect of physical exercise on regulatory T cell homeostasis by increasing their cell counts and anti-inflammatory potential [9].

Moreover, there are emerging biopsychosocial models that link a dysregulated immune system, i.e. proinflammatory cytokines, to cognitive impairment and fatigue which represent the most relevant symptoms for pwMS [10, 11]. It was shown in different patient cohorts that exercise might be a promising therapeutic approach to improve cognitive performance [12–14] with both resistance and endurance exercise being feasible and beneficial for pwMS [15, 16].

The classification of MS as an autoimmune neuroinflammatory disease favored research to elucidate how activated peripheral (autoreactive) immune cells or neurotoxic substances invade into the central nervous system (CNS) and contribute to disease progression. Increased secretion of proteolytic matrix metalloproteinases (MMP), i.e. MMP-2, as modulators of blood-brain barrier (BBB) integrity seems to have profound implications for MS [17–19]. We have shown that, in contrast to moderate continuous training, high intensity interval training over a period of 3 weeks reduces serum levels of MMP-2 [20]. Further, a recent systematic review of human intervention studies concludes that exercise training positively modulates BBB permeability markers in pwMS [21].

Acute intense exercise provokes a transient inflammatory state and enzymatic conversion of tryptophan (TRP) to kynurenine (KYN) and further KYN metabolites ( [22], Joisten 2019 accepted). It is assumed that exercise-induced activation of immune cells plays a significant role in this process. KYN may has potent immune regulatory characteristics by inhibiting the activation of pro-inflammatory immune cells while promoting differentiation of regulatory T cells [23, 24]. However, chronic synthesis of KYN through long term peripheral immune dysregulation seems to be involved into the pathophysiology of MS [25, 26]. By passing the BBB, KYN can be metabolized into neurotoxic quinolinic acid (QUIN) by locally activated immune cells. Exercise-induced conversion of KYN to kynurenic acid (KYNA) and NAD+ may decrease translocation of KYN into CNS, thereby limiting kynurenine-associated neurotoxicity [27, 28].

In a review discussing the relevance of immune regulatory treatments in MS, Dendrou et al. [1] recommend multimodal targeting of peripheral and CNS-intrinsic inflammation in early disease stages. Further, Riemenschneider et al. [29] propose to conduct exercise treatments in the early disease stage to promote evidence-based clinical integration for a “window of opportunity” in MS exercise therapy.

Since opportunities for early-phase MS rehabilitation in rehabilitation centers are rare and limited to some weeks per year, the overall objective of this study is to investigate the chronic effects of a community-based 12-week exercise training in a publicly available setting in pwMS with light symptomatology. By correlating the assessed cognitive capacity and patient-reported outcomes like anxiety/depression and fatigue with immune-related biomarkers and cell populations, existing hypothetical biopsychosocial models in pwMS [10] will be addressed in the context of exercise neuroimmunology.

## Methods/design

In this study, a two-armed single-blind randomized controlled trial design will be applied. Prior to (T_0_) and 48 h after the intervention period (T_2_), an assessment of cardiovascular fitness, muscular strength, cognitive performance, gait performance and patient-reported outcomes (depression and anxiety, fatigue, quality of life, sleep quality, physical activity behavior, social integration) will be conducted. Blood samples at rest will be taken at T_0_, midpoint (T_1_) and T_2_. To investigate acute changes of BBB markers, immune homeostasis, (anti-) inflammatory factors as well as TRP metabolites, there will be an additional assessment of blood samples before (T_1_), during (T_1.1_) and after (T_1.2_) a training session with the resting values at T_1_ being further used for midpoint follow-up. Blood samples from the control group at T_1_ will be assessed without further measurements at T_1.1_ and T_1.2_. To control for the participants’ daily activity level, the participants will be asked to adhere to a 7-day actigraphic measurement on three time-points (T_0_,T_1_,T_2_).

All assessments and blood-sampling will be conducted at the German Sport University Cologne, except for the blood samples at T_1_,T_1.1_,T_1.2_ in the intervention group. This blood sampling will be done by a physician at the fitness center.

Due to the nature of the trial, participants could not be blinded to group allocation. Assessors taking outcome measures post intervention will be blinded to group allocation. The training sessions will be supervised by MS experienced exercise scientists. The trainers will not be involved in the post assessment to keep blinding.

### Participants and recruitment

A total of 64 persons with definite diagnosis of relapsing-remitting multiple sclerosis (RRMS) (revised McDonald criteria [30]) will be recruited through announcements at the German Sports University Cologne, the Deutsche Multiple Sklerose Gesellschaft (DMSG, Ortsvereinigung Köln und Umgebung e.V.), and local MS clinics. The participants will be screened for eligibility by the study supervisor with further key inclusion criteria of an Expanded Disability Status Scale (EDSS) [31] score between 0 and 4.0.

### Eligibility

Prior to inclusion, participants will be screened for exclusion criteria and general sports capability (see Table 1). If eligible, the participant will be asked to provide written informed consent. After stratification, the participants will be randomized to either the intervention group or a passive waitlist control group.

**Table 1:** Detailed list for eligibility screening

Inclusion criteria	Exclusion criteria
♦ *Age: 18–45 years*	♦ *Acute episode or significant exacerbation of multiple sclerosis symptoms during intervention*
♦ *EDSS: 0–4.0 (inclusive)*	
♦ *Approved sport capability*	
♦ *Approved RRMS*	♦ *Cardiac preload*
	♦ *Orthopaedic diseases limiting the execution of the intervention and assessments*
	♦ *Problems in understanding (not German-speaking)*
	♦ *Concomitant disease states (internistic or neurological) affecting study outcomes*
	♦ *Cancer diseases under treatment of chemotherapy or radiation therapy*
	♦ *Pregnant or breast-feeding women*

*EDSS* Expanded Disability Status Scale, *RRMS* Relapsing-remitting multiple sclerosis

### Randomization

Participants will be randomly allocated (1:1) to either the intervention group or a passive waitlist control group. A concealed randomization approach will be conducted by an independent employee using “Randomization-In-Treatment-Arms” software (RITA, Evident, Germany) that follows the minimization procedure [32]. To ensure equal allocation of subgroups of participants to both experimental conditions, following stratification factors will be used: age, lean body mass, disease severity (EDSS), intake of immune regulatory medication, the sum of the hypothetical one-repetition maximums (h1RM_sum_) and peak power output (Watt_max_).

### Study intervention

#### Experimental intervention group

All training sessions will take place in a publicly available fitness center in Cologne, Germany. Participants of the intervention group will conduct three training sessions per week for a period of 12 weeks. In groups of maximal ten, the participants will perform endurance and resistance exercises for 60 min with 30 min of resistance exercises and 20 min of endurance/ strength endurance exercises. Each session will be supervised by MS experienced exercise scientists. To guarantee appropriate muscle activation, a 10-min warm-up with light aerobic activity and mobilization will be applied. An additional table shows the exercise program in more detail (see Additional file 1). To avoid difficulties with fixed-time training sessions and to improve adherence, participants will be able to choose between different weekdays and time slots.

A progressive periodization with step by step learning will be applied during the first 2 weeks of the intervention to guarantee precise movement execution of free weight resistance exercises. Thereafter, the participants will increase the load in consultation with the trainer.

The endurance / strength-endurance part comprises alternating short ergometer exercises, body weight exercises and exercises with small, functional devices (e.g. medicine ball or kettlebell) with rest periods of at least 1 min after each exercise. The exercises follow the principle “as much repetitions as possible”. The workload will be increased from 4 × 2 min to 4 × 3 min to 4 × 4 min according to the performance level of the participants.

#### Passive control group

**Fig. 1:**
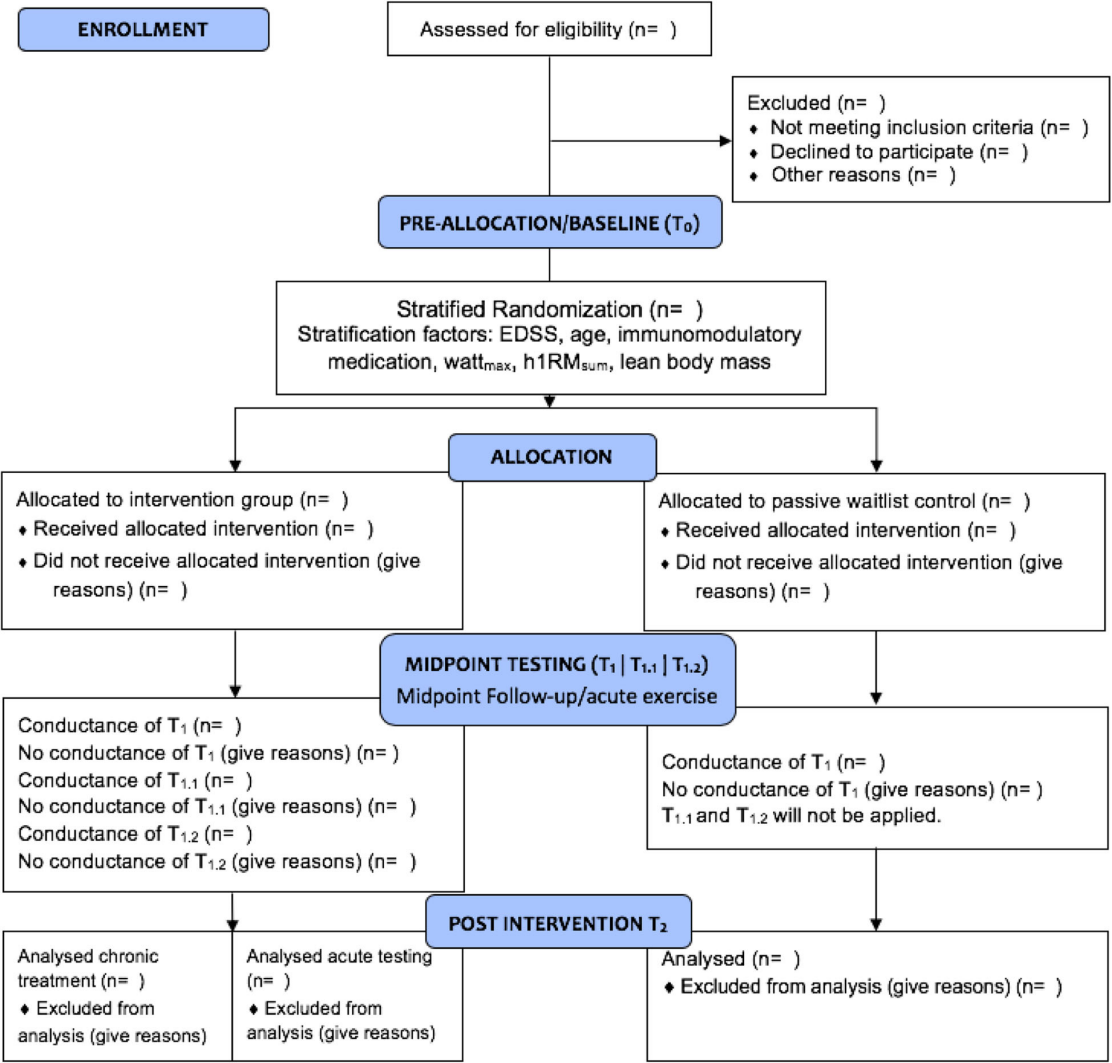
Flow diagram of this study. Baseline testing (T_0_) will be done before stratified randomization/allocation to either group. There will be a further collection of blood samples 6 weeks after intervention onset (midpoint) at rest (T_1_), after resistance exercises (T_1.1_) and immediately after the training session (T_1.2_) to investigate effects of acute training. Assessment of blood samples from the control group at T_1_ will be done at the German Sport University Cologne without further measurements at T_1.1_ and T_1.2_. After the 12-week intervention period (T_2_), all outcome measures will be assessed 48 h after the last training session to investigate effects of chronic training

For ethical reasons, it is not justifiable to withhold the exercise intervention completely from the passive control group. Therefore, the study controls will be included in a waiting list to get offered the same exercise program after T_2_ (Fig. 1).

### Outcomes and assessments

#### Primary outcome

**Fig. 2:**
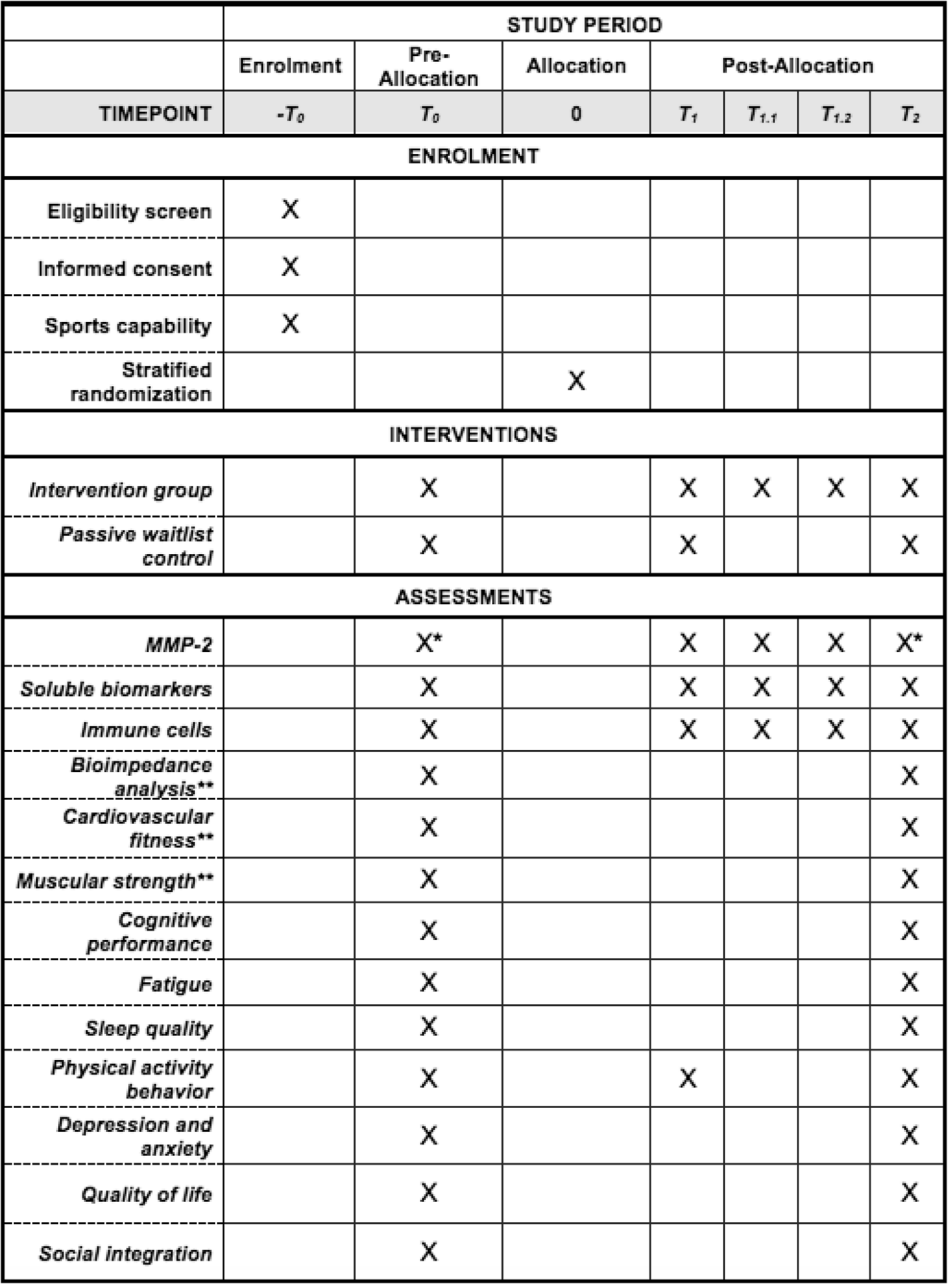
Spirit diagram depicting the schedule of enrolment, interventions and assessments. T_0_ = baseline assessment before stratified randomization; T_1_ = collection of blood samples immediately before a training session at midpoint (6 weeks after intervention onset). Biomarkers at T_1_ will be further used for midpoint follow-up. Blood samples from the control group will be assessed at the German Sport University Cologne without further measurements at T_1.1_ and T_1.2_; T_1.1_ = collection of blood samples after resistance exercises; T_1.2_ = collection of blood samples immediately after the training session; T_2_ = assessment of outcomes 48 h after the last exercise session; * primary outcome; ** stratification factors: i) Bioimpedance analysis using lean body mass; ii) Cardiovascular fitness using Watt_max_; iii) Muscular strength using h1RM_sum_

The primary aim of this study will be to examine the effect of a 12-week exercise intervention period on serum concentrations of MMP-2 compared to control (see Fig. 2). We hypothesize that the decrease in MMP-2 will be greater in exercising MS patients compared with control.

Blood samples will be taken by qualified technicians via vein puncture from the antecubital vein in supine position at T_0_ and T_2_ at the German Sport University Cologne. Blood samples will be centrifuged at 3000 g for 10 min at 4 °C, and the supernatant will be stored at − 40 °C until study completion. MMP-2 will be determined by enzyme-linked immune sorbent assay (ELISA) (R&D Systems, Inc., Minneapolis, MN, USA) according to the manufacturer’s instructions.

#### Secondary outcomes

Blood samples to determine chronic (T_0_ to T_2_) and acute (T_1_,T_1.1_,T_1.2_) changes of biological secondary outcomes, i.e. immune cell subsets and soluble (anti-) inflammatory factors, will be collected at the same time as those of the primary outcome (see Fig. 2). Serum will be stored at − 80 °C until analysis after study completion. The assessment of physical and cognitive performance, patient-reported outcomes, body composition and biomechanical motion analysis will be done at T_0_ and T_2_.

### Immune cell counts

Numbers and proportions of T cells (CD3^+^), T-helper cells (CD3^+^CD4^+^), Th17 cells (CD3^+^CD4^+^CD161^+^CCR6^+^), and regulatory T cells (CD3^+^CD4^+^CD25^+^CD127^dim^) will be assessed at all time points (chronic (T_0_,T_2_) and acute (T_1_,T_1.1_,T_1.2_)) and determined by using flow cytometry.

Gating strategies will be applied as reported by Zimmer et al. and Wenning et al. [33, 34].

### Soluble factors

Inflammatory (MMP-9, Tumor necrosis factor-alpha (TNFα), Interferon gamma (IFNγ), Interleukin (IL)-6, IL-17) and anti-inflammatory (IL-10) soluble factors as well as TRP and metabolites (KYN, KYNA, QUIN, NAD+) will be assessed at all time points. Serum MMP-2 will be assessed at T_1_, T_1.1_ and T_1.2_ as well for investigating acute serum changes after a single training session.

Serum levels of TRP, KYN, TNFα, IFNγ, IL-6, MMP-9, IL-10 and IL-17 will be determined by using ELISA according to manufactures instructions. Levels of KYNA and QUIN will be assessed by high-performance liquid chromatography. Further, enzymes involved into the TRP degradation pathway and KYN metabolism (Kynurenin-3-Monooxygenase, Indolamin-2,3-Dioxygenase, Kynurenin-Aminotransferase) will be measured by real-time quantitative PCR.

Serum levels of neurofilament light chain (NfL) will be measured by the single-molecule enzyme-linked immunosorbent assay (ELISA) called single molecule array (Simoa). That technology was shown to be 126- and 25-fold more sensitive than regular ELISA or electrochemiluminescence, respectively [35]. Measurement will be done as described previously by Disanto et al. [36].

### Assessment of physical capacity

To assess body composition of the participants, bioelectrical impedance analysis will be used and recorded according to the manufacturer’s instructions. The calculated lean body mass will be used as a stratification factor for randomization, since visceral adipose tissue has been described to play a significant role in inflammatory signalling [37] and therefore, may impact biological outcome measures.

Cardiorespiratory fitness will be assessed on a cycle ergometer (Ergoline®, Bitz, Germany) with a progressive cardiopulmonary exercise test (CPET). Participants will begin the test at an initial intensity of 30 W (one-minute warm-up), increasing by 15 W every minute until volitional exhaustion. This will be followed by a three-minute cool-down. The resulting Watt_max_ at T_0_ will be used as a stratification factor for randomization. The individual cardiorespiratory fitness level will be monitored by direct and continuous measurements (breath by breath) of VO_2peak_ by ergospirometry (Cortex, MetaLyzer 3B-R2, Germany). Blood lactate will be measured before, immediately after and 3 min after the CPET via earlobe prick.

To assess muscular strength, a five-repetition maximum (5RM) test will be conducted for lower (leg press) and upper body compartments (chest press, lat pulldown). The American College of Sports Medicine (ACSM) guidelines will be applied to assess the 5RM [38]. These guidelines state that, firstly, the participants should warm up by completing several submaximal repetitions. Secondly, the 5RM will be determined within four trials with rest periods of 3 min between trials. Thirdly, an initial weight that is within the subject’s perceived capacity (~ 50–70% of capacity) will be chosen. Thereafter, the resistance will be progressively increased by 2.5-20 kg until the subject is not able to complete the selected repetition(s). All repetitions should be performed at the same speed of movement and range of motion to ensure consistency between trials. Lastly, the final weight lifted successfully as the absolute 5RM will be recorded.

The rest periods between training sets will be decreased to 2 min according to Miller [39] and Brown [40]. Following instructions from Landers et al. [41], the hypothetical one-repetition maximum (h1RM) will be calculated from the 5RM test results. The calculated h1RM of the different exercises are added together with the sum representing the outcome measure (h1RM_sum_) for muscular strength and will be used as a stratification factor.

### Motion capture

As maintenance of safe locomotion is important in the course of MS, a biomechanical motion analysis using a Vicon System (Vicon, Oxford, UK) and three-dimensional force plates (Kistler, Winterthur, Schweiz) will be applied. By using an inverse-dynamic model the mechanical power on the ankle as well as the absorption and the release of energy will be captured as parameters in addition to the ground reaction forces.

### Assessment of cognitive performance

To test the cognitive performance of the participants, the Brief International Cognitive Assessment for Multiple Sclerosis (BICAMS) battery [42] will be applied. The battery comprises three tests to assess information processing speed, verbal memory and visual memory which all constitute major cognitive domains affected in the course of MS.

Information processing speed will be measured with the Symbol Digit Modalities Test (SDMT). Digits from one to nine are each paired with one of nine abstract symbols, with a correct assignment being visible for the participant throughout the testing. There is a pseudo-randomized sequence of a total of 110 symbols on a working sheet. After a training part of pairing ten symbols is completed, the participant responds by saying the corresponding digits of the remaining symbols as quickly as possible. The indicator of performance is the total number of correct symbol-digit pairs in 90 s.

Verbal memory will be measured with the California Verbal Learning Test (CVLT) that shows good external validity in MS [43]. The German version will be applied according to Niemann et al. [44]. The test contains two 16-items word lists (list A, list B) of which just list A will be used in this study. The list will be read out loud by the investigator. Thereafter, the participant will be asked to recall as many words as possible without any time restriction. This procedure will be repeated in five consecutive trials. The indicator of performance is the total number of words recalled during all trials.

Visual memory will be measured with the Brief Visuospatial Memory Test-Revised (BVMT-R). Six abstract geometric figures will be shown to the participant for 10 sec in three consecutive trials. After the presentation of the visual stimulus figures, the participant will be asked to draw, without any time restriction, as many recalled figures as possible in the correct location, shape and proportion. Each figure will be scored 0, 1, or 2 based on the scoring criteria. The indicator of performance is the total recall score in all three trials.

To exclude memory effects regarding verbal and visual memory, parallel versions of the CVLT and BVMT-R will be applied at T_2_.

### Patient-reported outcomes

To assess physical activity behavior, both subjective and objective measures will be applied three times (T_0_,T_1_,T_2_).

For subjective measurement, the Godin Leisure-Time Exercise Questionnaire (GLTEQ) and the activity questionnaire will be used, respectively. The GLTEQ asks for weekly frequencies of strenuous (9 points), moderate (5 points), and mild (3 points) activities with all points being multiplied by the number of weekly conductance. Reference score units are ≥24 (≙ active), 14 to 23 (≙ moderately active) and < 14 (≙ insufficiently active) [45].

The second activity questionnaire is asking for the activity of the patients during the last 1, 6 and 12 weeks using either i) boxes to provide exact time of physical activity (in hours) and exercise (in minutes) during the last week and ii) visual analogue scales ranging from “low activity” to “high activity” to assess physical activity in the last 1, 6 and 12 weeks.

For objective measurement, an actimetry sensor (ActiGraph GT9X Link) is applied to each participant for seven consecutive days in order to identify differences in rest and activity cycles. The sensor will be attached to the hip to better monitor the activity level. There will be several interesting outcome measures with moderate to vigorous physical activity (MVPA) being the main outcome measure.

Fatigue will be assessed with the two-dimensional Fatigue Scale for Motor and Cognitive functions (FSMC). The FSMC has defined cut-off scores to differentiate between mildly (≥43 sum score), moderately (≥53 sum score) and severely (≥63 sum score) fatigued patients using 20 items with ten items for each dimension [46]. The German version shows excellent test-retest reliability [47].

Depression and anxiety will be measured with the German version of the Center for Epidemiologic Studies Depression Scale (CES-D) [48, 49]. In a 20-item measure using a four-point Likert scale, the participants will be asked to rate how often they experienced symptoms associated with depression over the past one to 2 weeks.

Health-related quality of life will be evaluated with the Multiple Sclerosis International Quality of Life Questionnaire (MusiQol) by describing nine dimensions with 31 items [50]. The German version shows good test-retest reliability [51].

Sleeping quality will be assessed by the German version of the Pittsburgh Sleep Quality Index (PSQI) [52]. Participants rate their subjective sleep quality, sleep latency, sleep duration, habitual sleep efficiency, sleep disturbances, use of sleeping medication, and daytime sleepiness over the past 4 weeks.

Social integration will be assessed with the German version of a social integration index according to Bittner et al. [53]. The index contains three domains, each scored from 0 to 2 (total range of index from 0 to 6) [54]. Level I of the index includes persons who scored 0 or 1. Further levels are II (range 2–3), III (range 4–5), and IV (6). The index includes three types of ties: 1) marital status or cohabitation, 2) contacts with close friends and family, and 3) affiliation with voluntary associations. Marital status will be scored 0 if the subject is single/divorced/widowed and 2 if the subject is married/living with a partner. Regarding contacts, subjects are given a score of 0 (0–2 contacts), 1 (3–11 contacts), or 2 (≥12 contacts). The affiliation with voluntary associations be calculated by positive responses to questions on membership in any of six types of political, religious, community, sports, or professional organizations.

All questionnaire-based patient-reported outcomes will be assessed using a tablet computer (iPad, 128GB Version 2018). For this purpose, Qualtrics Research Core software will be applied.

### Data management

In order to monitor participant adherence to the weekly training sessions, a training sheet will be implemented that will i) show whether intervention group participants took part in the planned training sessions and ii) be used to document training progression. Participants will be contacted by email or phone if they fail to take part in two consecutive training sessions.

Additionally, as a safeguard for any potential data loss, all study data will be stored in a password-protected computer device that is not connected to the internet. Additionally, training documentation data of each participant will be entered in the computer device on a weekly basis to provide fast and easy access to training adherence and training progression.

### Data analysis

Statistical analyses will be performed by a statistician blinded to treatment groups. Level of significance is set at *p* ≤ 0.05. All values will be presented in means ± standard deviation. Statistical analyses will be conducted using the actual SPSS Version® (IBM®, Armonk, NY, USA) and R.

The required sample size to evaluate the between-group effect of the intervention on the primary outcome (serum changes of MMP-2 from baseline (T_0_) to post (T_2_)) in an analysis of covariance model (ANCOVA) was estimated using G*Power 3.1.9.2 [55]. Serum concentration of MMP-2 at baseline (T_0_) will be used as covariate. Based on already published study results [20], a medium to large effect size (Cohens d = 0.6) was used for sample size calculation. Statistical significance will be set 5% with statistical power (1-β) of 0.8. The sample size was calculated according to the formula N = (1-r2)*n described by Borm et al. [56]. The correlation was conservatively set at r = 0.6 which results in a preliminary sample size of *N* = 58. The expected drop-out rate is 15%. Therefore, a total of 64 participants will be recruited.

### Primary analysis

An intention-to-treat analysis will be conducted. To investigate the effect of the intervention (12-week exercise intervention of combined functional resistance and endurance exercise) compared to control on the primary outcome MMP-2, we will conduct a 2 (intervention group vs. control group) × 2 (T_0_ vs. T_2_) analysis of covariance (ANCOVA). This model will be adjusted for baseline values and lean body mass at T_0_. As measure of effect size partial Eta-squared and Cohen’s d values, respectively, will be calculated.

### Secondary analysis

An intention-to-treat analysis will be conducted. To investigate the effect of the intervention compared to control on secondary outcomes, a baseline-adjusted 2 (intervention group vs. control group) × 2 (T_0_ vs. T_2_) ANCOVA model will be applied. To determine if groups differ at T_1_ and/or at T_2_, simple effects analyses (Bonferroni corrected) will be performed.

As measure of effect size partial Eta-squared and Cohen’s d values, respectively, will be calculated. Bivariate correlation analyses (Pearson) will be conducted to determine potential associations between changes in biomarkers and physical performance outcomes, cognitive performance and/or patient-reported outcomes from T_0_ to T_2_ (delta T_2_-T_0_).

To investigate the effect of an acute training session on biological markers in intervention group participants, we will conduct a repeated measures ANOVA at T_1_ (baseline), T_1.1_ (during) and T_1.2_ (post) exercise session.

### Safety

To ensure safety during each training session, exercise intensity will be monitored both objectively and subjectively based on the participants heart rate (measured by Polar FS1C) and their rating of perceived exertion (RPE, Borg scale (6-20)), respectively. Further, there will be a training sheet that will be filled in by the participants during each training session to monitor adequate training progression and intensity. The MS experienced exercise scientist who will conduct the training will contribute substantially to the safety of each participant as well. All trainers are able to administer first aid.

All severe adverse events – that include i) severe cardiovascular and pulmonary diseases (e.g. renal failure, hepatic dysfunction, cardiovascular disease) and ii) severe cardiovascular exacerbations (e.g. RR > 240/120) during a training session will be directly reported to the ethical committee.

The drop-out rate will be recorded in total and separately for each group.

## Discussion

Disability in pwMS often continues to worsen despite immunotherapy, so alternative and/or adjunct treatments seem imperative for this patient cohort [1]. Besides the need of encouraging pwMS to engage more in moderate to vigorous exercise [6], existing (multimodal) therapeutic exercise regimens need to be continuously optimized in order to decrease the socioeconomic consequences of MS and to improve the quality of life of pwMS. In this study only persons with a relatively low disease severity (EDSS 0–4) will be included. Due to the fact that the disease-specific symptomatology is limited only to a few systems, the probability to include participants in an early disease state is increased. That in turn allows to take advantage of a proposed “window of opportunity” that may be a promising approach to effectively slow down disease progression [29].

Moreover, the approach of this study, i.e. community-based training with combined functional resistance and endurance exercises in a publicly available fitness center, may have profound impact on general feasibility of effective exercise treatments and optimization of existing exercise recommendations.

Improving the BBB integrity by reducing MMP-2 serum concentration as the primary outcome of this study and to establish immune homeostasis seem to be an integral component for decreasing both MS-specific symptomology and disease progression.

Another important objective of this study is to test assumptions of existing hypothetical biopsychosocial models in pwMS [9, 10] in the context of exercise neuroimmunology by correlating the assessed cognitive capacity and patient-reported outcomes like anxiety/depression and fatigue with immune-related biomarkers and cell populations. Moreover, the assessment of the acute effect will extend knowledge of acute exercise-induced effects on immune signaling, proportions on immune cell subsets and the kynurenine pathway in persons with RRMS.

## Supplementary information

**Additional file 1.** Design of the 60 min training sessions. * SkiErg, rowing machine; ** during the first 2 weeks: technical introduction and habituation to training with free weights; thereafter: progressive increase in weights with one resistance exercise for the lower body and one for the upper body 15 min each in one workout; *** rope, light barbells etc.

## Data Availability

Not applicable.

## Abbreviations

5RM: Five-repetition maximum
ACSM: American College of Sports Medicine
ANCOVA: Analysis of Covariance
BBB: Blood brain barrier
BICAMS: Brief International Cognitive Assessment for Multiple Sclerosis
BVMT-R: Brief Visuospatial Memory Test revised
CD: Cluster of differentiation
CES-D: Center for Epidemiologic Studies Depression Scale
CNS: Central nervous system
CPET: Cardiopulmonary exercise test
CVLT: California Verbal Learning Test
DMSG: Deutsche Gesellschaft für Multiple Sklerose
EDSS: Expanded disability status scale
ELISA: Enzyme-linked immunosorbent assay
FSMC: Fatigue scale of motor and cognitive functions
GLTEQ: Godin Leisure-Time Exercise Questionnaire
h1RM: Hypothetical one-repetition maximum
IFNγ: Interferon gamma
IL: Interleukin
KYN: Kynurenine
KYNA: Kynurenic acid
min: minutes
MMP: Matrix metalloproteinase
MS: Multiple sclerosis
MusiQol: Multiple Sclerosis International Quality of Life Questionnaire
NfL: Neurofilament light chain
PSQI: Pittsburgh Sleep Quality Index
pwMS: persons with MS
QUIN: Quinolinic acid
RPE: Rating of perceived exertion
RRMS: Relapsing-remitting multiple sclerosis
SDMT: Symbol Digit modalities test
Simoa: single molecule array
TNFα: Tumor necrosis factor-alpha
TRP: Tryptophan
VO2_peak_: Peak oxygen consumption
