# Supplementary figures and images for: Influence of combined functional resistance and endurance exercise over 12 weeks on matrix metalloproteinase-2 serum concentration in persons with relapsing-remitting multiple sclerosis – a community-based randomized controlled trial
Source: BMC Neurol. 2019 Dec 6;19:314. doi: 10.1186/s12883-019-1544-7 (PMC6898928; doi:10.1186/s12883-019-1544-7)

Additional file 1


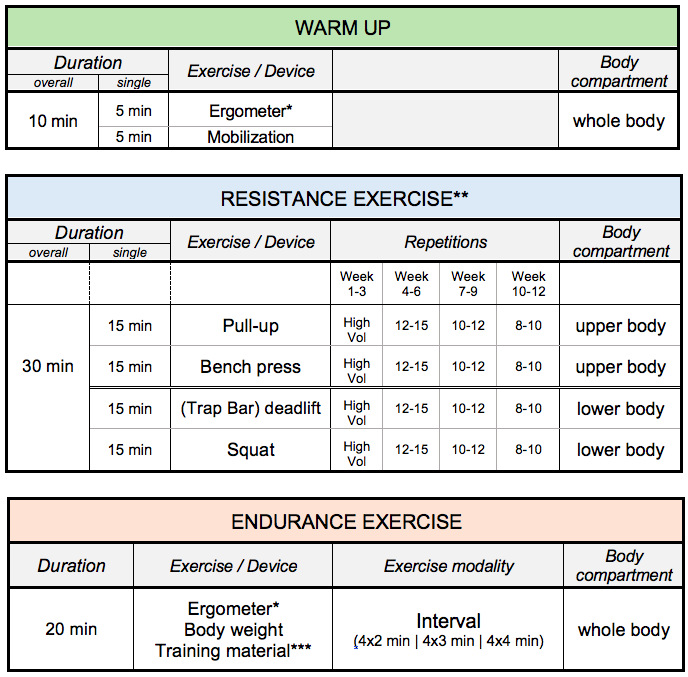

Supplement: Supplementary file 1 — Additional file 1. Design of the 60 min training sessions. * SkiErg, rowing machine; ** during the first 2 weeks: technical introduction and habituation to training with free weights; thereafter: progressive increase in weights with one resistance exercise for the lower body and one for the upper body 15 min each in one workout; *** rope, light barbells etc. [file 12883_2019_1544_MOESM1_ESM.docx]
